# NRAS Gene Mutation in Differentiated High-Grade Thyroid Carcinoma With Multiphenotypic Metastatic Pattern: A Case Report

**DOI:** 10.7759/cureus.32177

**Published:** 2022-12-04

**Authors:** Juan C Alvarez Moreno, Palawinnage Muthukumarana, Suimin Qiu

**Affiliations:** 1 Pathology, University of Texas Medical Branch, Galveston, USA; 2 Pathology and Laboratory Medicine, University of Texas Medical Branch, Galveston, USA

**Keywords:** lymph node metastasis, bone metastasis, follicular carcinoma of the thyroid, papillary carcinoma of thyroid, nras, thyroid pathology

## Abstract

Differentiated high-grade thyroid carcinoma (DHGTC) has a high mitotic count (≥5 mitoses per 2mm^2^) and/or tumor necrosis without anaplastic features. These tumors are rare, and the prevalence is not yet established among thyroid malignancies. BRAF andRAS mutations are the main driver mutations in these tumors. We present a case of a 43-year-old woman with DHGTC and NRASmutation, presenting with metastatic follicular component to the bone and papillary component to lymph nodes.

## Introduction

The WHO defines follicular-derived carcinomas, high-grade, as carcinomas of thyroid follicular cells that can be poorly differentiated or retained morphology of well-differentiated carcinomas, with high-grade features defined by mitotic count and tumor necrosis without anaplastic histology [1]. The prevalence varies from 1% to 6.7% [2,3]; the incidence in North America is 1.8%, the mean age is 55-65 years [4], and it is more common in females than in males. It is rare in the pediatric population. This group of neoplasms has two subtypes, poorly-differentiated thyroid carcinoma (PDTC) and differentiated high-grade thyroid carcinoma (DHGTC). The Turin consensus defines PDTC as a solid, trabecular, or insular growth pattern; absence of nuclear features of papillary thyroid carcinoma; and at least one of the following features - convoluted nuclei, mitotic index of ≥3/10 high-power fields, and/or tumor necrosis [5]. Memorial Sloan Kettering Cancer Center defined PDTC as elevated mitotic index ≥5/10 HPFs and/or tumor necrosis but with well-differentiated thyroid histology [6]. The WHO defines DHGTC as well-differentiated thyroid carcinomas with increased mitotic counts and/or tumor necrosis. The mitotic count must, by definition, be ≥5 mitoses per 2 mm^2^ on hot spots.

We present a case report of a DHGTC, presenting with metastatic follicular component to the bone and papillary component to lymph nodes. Molecular studies showed oncogenic NRAS mutation in all these areas, including the solid pattern of the primary thyroid carcinoma.

## Case presentation

**Figure 1:**
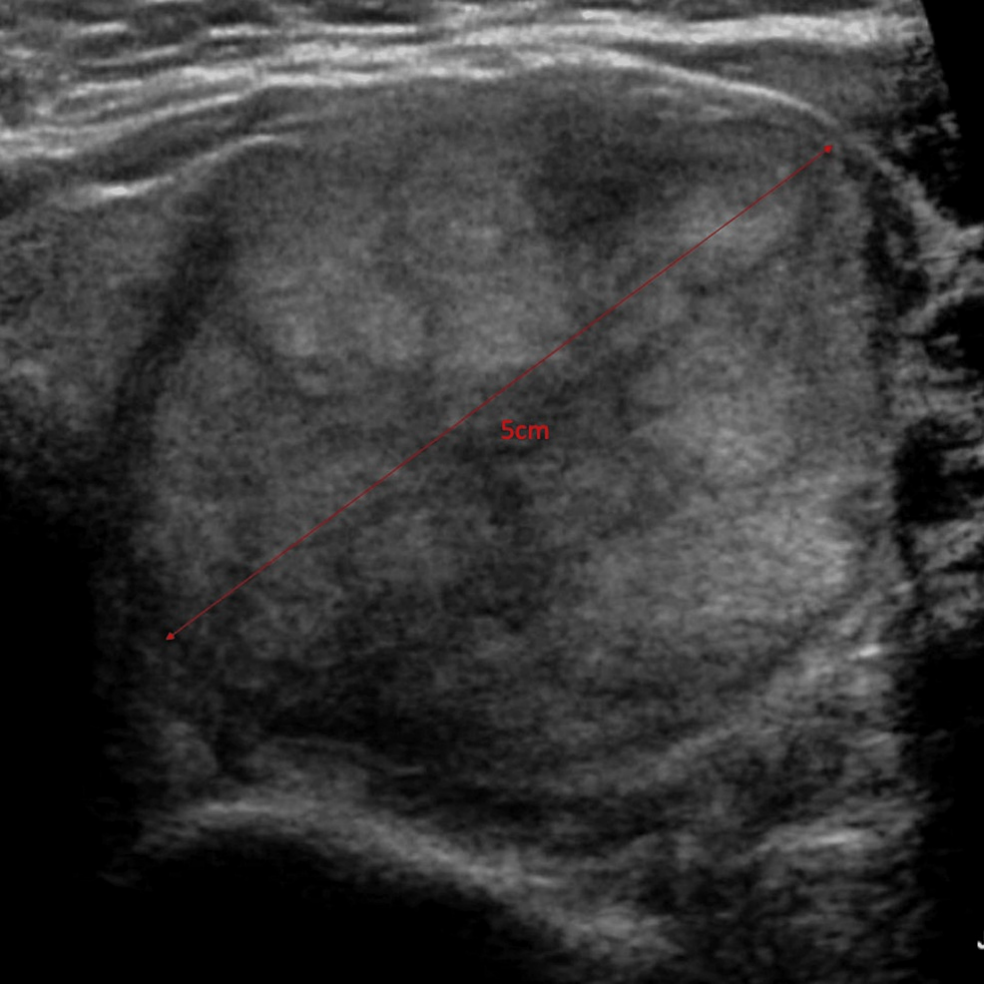
5.0 cm nodule on ultrasound

**Figure 2:**
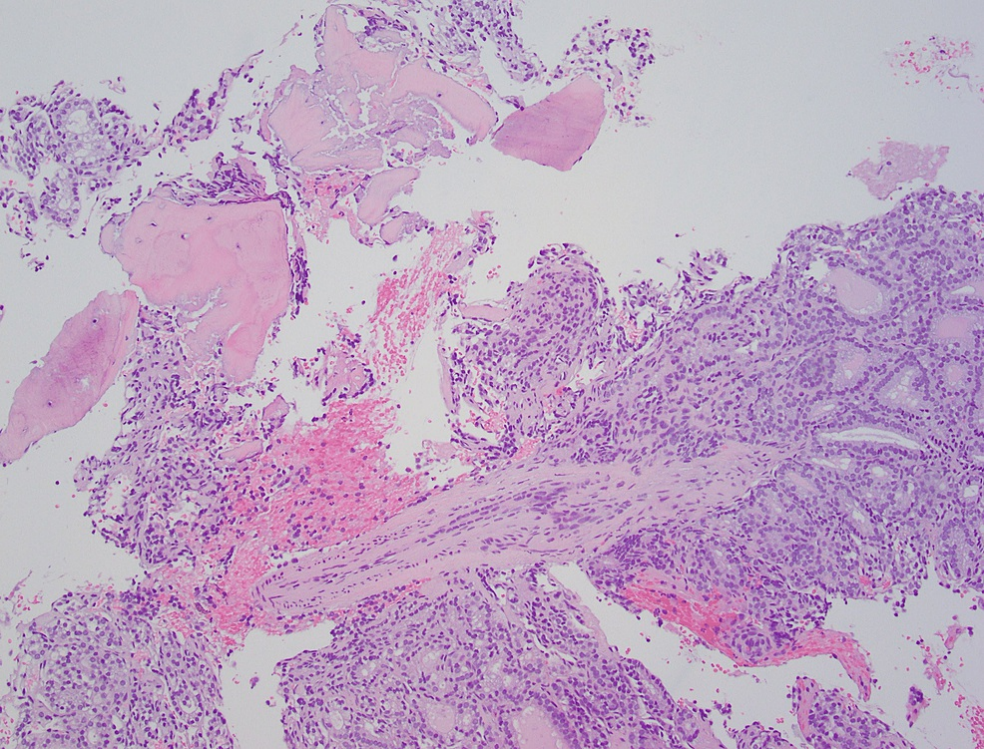
Histology of metastatic thyroid carcinoma with follicular morphology containing colloid, enlarged nuclei, pale chromatin, no grooves or pseudo-inclusions 10x

**Figure 3:**
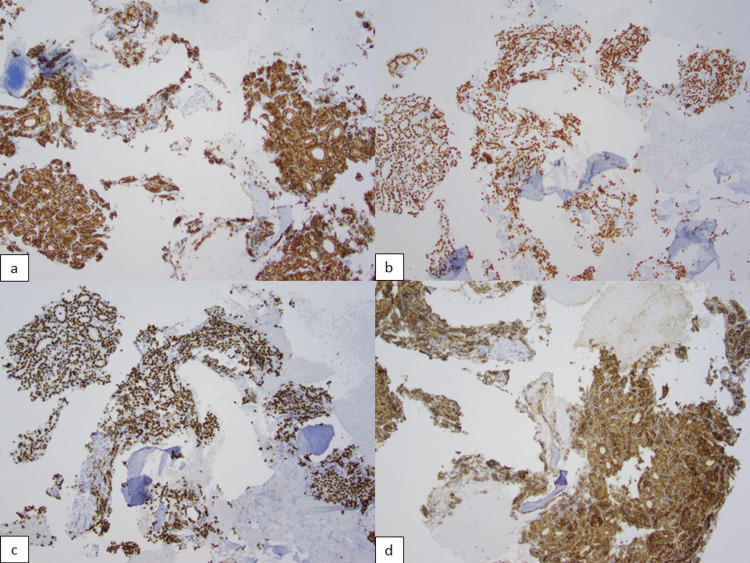
Metastatic follicular carcinoma immunohistochemistry profile: a) CK7 positive cytoplasmic immunostain 10x, b) TTF-1 positive nuclear immunostain 10x, c) PAX8 positive nuclear immunostain 10x, d) thyroglobulin cytoplasmic immunostain 10x

The patient is a 43-year-old woman with no significant medical history. She had no follow-up for a left thyroid nodule since 2017 that measured 5.0 cm (Figure 1) in greatest dimension in ultrasound. A fine-needle aspiration (FNA) of the nodule reported benign follicular nodule. No other imaging was done since the last ultrasound. She presented with dysphagia to our hospital. She denied any other signs and symptoms. A neck ultrasound revealed a TR4 (moderately suspicious) left thyroid nodule, which increased in size from 5 cm to 6.2 cm. CT of the neck reported an osteolytic mass of the T4 vertebral body with extraosseous extension. The vertebral body mass is biopsied, which showed follicles containing colloid admixed with bone fragments. The nuclei were enlarged with pale chromatin, and no grooves or pseudo-inclusions were identified (Figure 2). Immunohistochemical profile was CK7 positive (Figure 3a), TTF-1 strong positive (Figure 3b), PAX8 strong positive (Figure 3c), and thyroglobulin strong positive (Figure 3d), supporting a thyroid origin. We diagnosed it as metastatic thyroid carcinoma with morphological features of follicular carcinoma.

**Figure 4:**
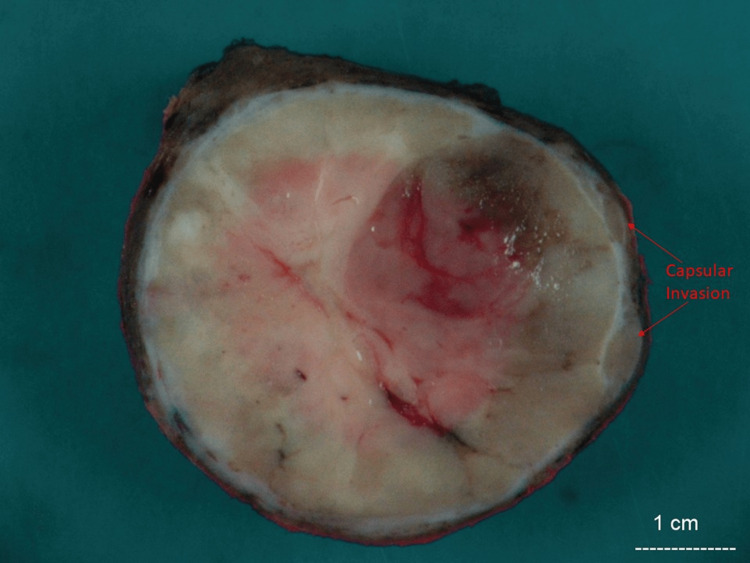
Gross picture of thyroid mass, 6.2 cm in greatest dimension with capsular invasion

**Figure 5:**
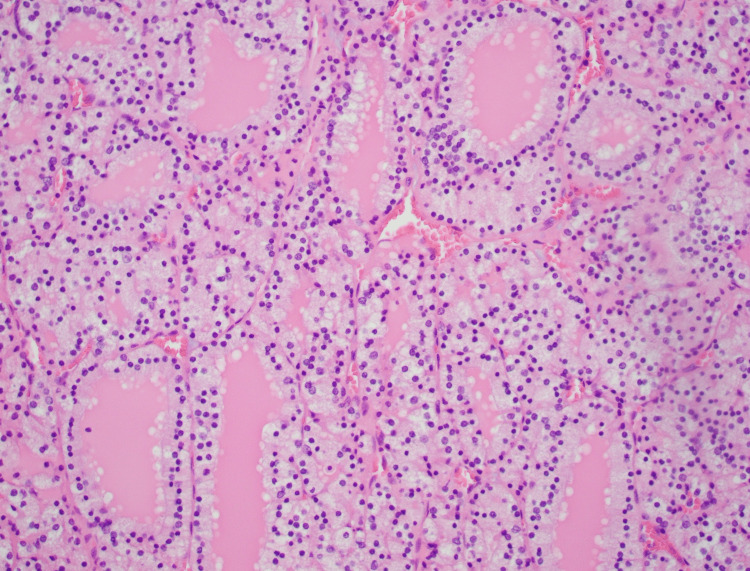
Histology of follicular pattern in thyroid carcinoma 20x

**Figure 6:**
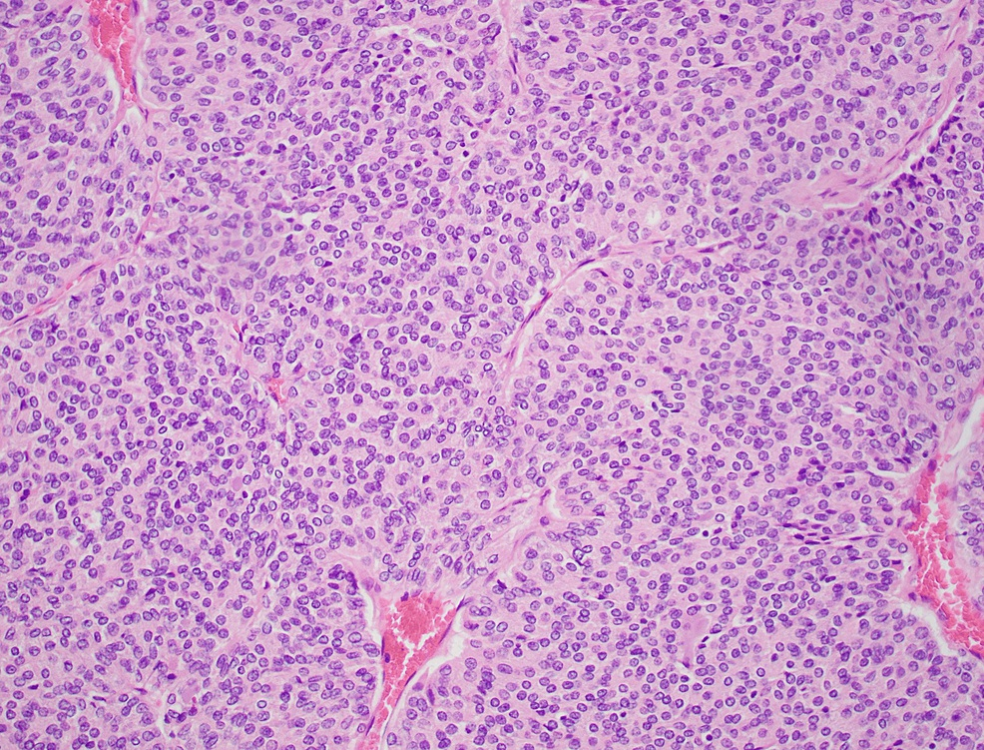
Histology of solid pattern in thyroid carcinoma 20x

**Figure 7:**
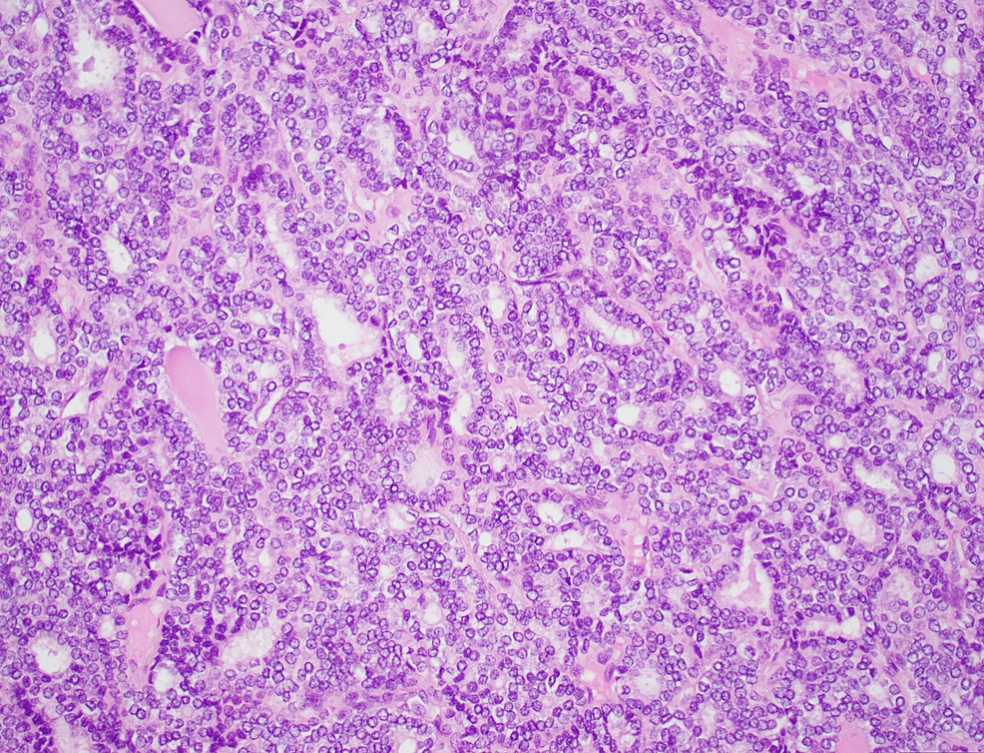
Histology of papillary thyroid carcinoma features showing nuclear crowding, clearing, enlargement, grooves, irregular nuclear membrane, and prominent nucleoli 20x

**Figure 8:**
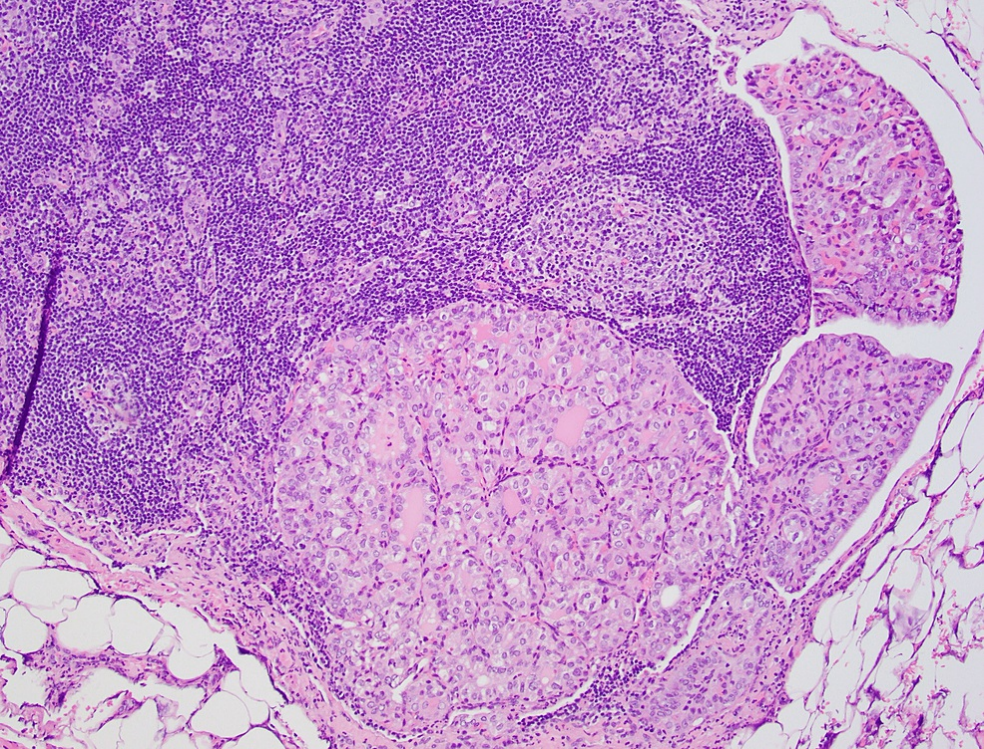
Histology of metastatic carcinoma with papillary thyroid carcinoma-like features to lymph node 10x

Total thyroidectomy with central neck dissection was performed on the patient. A gross examination of the specimen revealed a single encapsulated nodule in the left lobe with a heterogenous tan, soft pink cut surface with areas of capsular invasion (Figure 4). The nodule measured 6.2 x 5.0 x 4.4 cm. Histologically the tumor was composed of areas of varying morphological patterns ranging from small to normal-sized follicles with no papillary nuclear features (Figure 5) to solid architecture (Figure 6) and areas with large, crowed nuclei with clearing, nuclear enlargement, nuclear grooves, irregular membrane, and prominent nucleoli consistent with features of papillary thyroid carcinoma (Figure 7). The mitotic count was seven mitotic figures per 2mm^2^. Capsular invasion and extensive angiolymphatic invasion were identified. The metastatic clusters in three of fifteen lymph nodes display papillary thyroid carcinoma features (Figure 8). The BRAF immunostain was negative, and Ki-67 showed a proliferation rate of 7%. Molecular studies were performed on each of the different areas of the tumor, follicular, solid, and papillary, showing oncogenic NRAS mutations and negative for KRAS and BRAF mutations. The histologic profile alongside the mitotic index gives the diagnosis of differentiated high-grade thyroid carcinoma.

## Discussion

Differentiated high-grade thyroid carcinoma is uncommon. They usually present as a solitary mass growing over several months, sometimes in the background of a multinodular goiter. On an ultrasound scan, they are usually described as solid, heterogenous, and hypoechoic with irregular and indistinct borders [7]. Grossly on the cut surface, they are solid, brown to gray, with areas of necrosis. Although most of them are invasive, some may be partially or totally encapsulated [8]. Our tumor was encapsulated by imaging, as previously mentioned, and macroscopically and microscopically showed capsular invasion. The most common locations of distant metastases are the lung, bone, and brain [9]. Our case presented with bone metastases to the T4 vertebral body and lymph node metastases.

The pathogenesis of this tumor follows the progression of well-differentiated thyroid carcinomas retaining driving molecular signatures. BRAF and RAS mutations are the main driver mutations. According to the literature, these high-grade thyroid carcinomas gain additional mutations such as TERT, TP53, and PI3K [10,11,12]. Our case did not show any additional mutations. According to Xu et al.'s study [9], BRAF and NRAS mutations are mutually exclusive, but NRAS with E1F1AX or TERT mutations have concurrent alterations. Our case showed NRAS (c. 182A>G (p.Gln61Arg)) mutation in all histological patterns. Thyroid follicular carcinomas are known to harbor RAS mutation or PAX8/PPARy rearrangement [13]. The three RAS genes (HRAS, KRAS, and NRAS) encode highly related G-proteins that propagate signals arising in the cell membrane receptors to various intracellular targets. The mutations involving NRAS codon 61 and HRAS codon 61 are the most common [14]. The NRAS codon 61 is caused by the NRAS (A182G) mutation. Our case had the same NRAS mutation (c. 182A>G (p.Gln61Arg)), which is the most common in follicular pattern-based thyroid neoplasms. This mutation is related to hematogenous spread to bone and different histologic appearances [15,16]. These support our follicular pattern invading into the bone, as well as the solid areas in the primary. However, our case had papillary features invading into lymphatics and lymph nodes. Despite harboring the NRAS mutation, this pattern of invasion is extremely rare with this mutation. Although papillary thyroid carcinomas can have RAS mutations [17], the nuclear features are less prominent and have a low rate of lymph node metastases. According to Xu et al. [9], the disease metastasis-free survival in a five-year period is of 59%.

The neoplastic cells were positive for TTF1, thyroglobulin, keratins, and PAX8. According to Basolo et al. [15], the presence of NRAS mutation correlated with low expression of thyroglobulin expression. Recently a new immunohistochemical stain has been used to detect the NRAS Q61R antibody, which detects the same mutation as in our case, with a sensitivity of 90.6% and specificity of 92.3%, which can be helpful in follicular patterned neoplasms [18]. Although we did not use this antibody in our case, it will be ideal as a future surrogate biomarker for these neoplasms.

The differential diagnosis in our case is an unusual neoplasm, known as an encapsulated thyroid tumor of follicular cell origin with high-grade features. These tumors are defined as a follicular variant of papillary carcinoma with nuclear features of classical papillary thyroid carcinoma with a high mitotic rate (>5 mitoses/HPF) and tumor necrosis [8]. The most common mutation in this entity is theNRAS mutation, like in our case. They can present with capsular and vascular invasion, but lymphatic invasion or lymph node metastases are not common. What differentiates this neoplasm from our case is the presence of the follicular and solid pattern and lymph node metastasis.

## Conclusions

We present an interesting and unique case of multiphenotypic, metastatic NRAS-driven differentiated high-grade thyroid carcinoma. The papillary morphology metastasizing to a lymph node is a rare manifestation of this mutation compared to other reports in the literature. This case proves that despite the NRAS mutation, the papillary morphology of the tumor can still behave accordingly to its lymphatic predilection, while the follicular carcinoma component follows the hematogenous spread.
